# Therapeutic hypothermia in cardiac arrest survivors: is rebound hyperthermia a significant issue with intravascular cooling?

**DOI:** 10.1186/2197-425X-3-S1-A201

**Published:** 2015-10-01

**Authors:** T Price, C Poots, H Shields, R McKee

**Affiliations:** Department of Anaesthesia and Intensive Care, Craigavon Area Hospital, Craigavon, Northern Ireland United Kingdom

## Intr

Out-of-Hospital Cardiac Arrest (OOHCA) is associated with a poor prognosis. Targeted temperature management (TTM) within Intensive Care (ICU) including therapeutic hypothermia (TH) aims to reduce cerebral reperfusion injury and improve neurological outcomes.

Within Northern Ireland (NI), Craigavon Area Hospital (CAH) is the only ICU to implement TH using an intravascular cooling device (Coolgard 3000^©^, Alsius UK®)

The benefit of TH has recently been disputed and many ICUs within NI have since adopted TTM to 36°C in survivors of OOHCA [1].

In view of this we aimed to benchmark our use of TH to 32-34°C, using intravascular cooling against best practice at the time of data collection.

## Objectives

To assess:

· Demographics of patients receiving TH within CAH ICU

· Implementation, maintenance and temperature control during TH using intravascular cooling

· Outcomes of patients receiving TH

Against standards used in published reference journals [2, 3]

## Methods

Retrospective, observational chart-based data collection.

40 patients admitted to CAH ICU, who received TH via intravascular cooling catheter (24/5/2010-30/11/2012), were identified from the Intensive Care National Audit and Research Centre (ICNARC) database.

35 patients (87.5%) had available relevant and complete data.

## Results

**Table 1:** Indications for TH.

OOHCA with shockable rhythm	46%
OOHCA with non-shockable rhythm	23%
In-hospital cardiac arrest with shockable rhythm	11%
In-hospital cardiac arrest with non-shockable rhythm	8%
Respiratory Arrest	8%
Attempted suicide by hanging	4%

**Table 2:** TH using intravascular cooling.

Mean time from ROSC to target temperature of 32-34°C	8.3 hours
Mean duration of TH at temperature <34 °C	23.8 hours
Neuromuscular blockade use	28.5%
Rebound hyperthermia (>38°C) on cessation of active cooling	45.1%
Mean duration of rebound hyperthermia	8.6 hours

**Table 3:** Outcomes.

Mean length of ICU stay	5 days
ICU Mortality	45.7%
Destination at Hospital Discharge: Home	94.1%
Destination at Hospital Discharge: Hospice	5.9%
30 day mortality	60%

## Conclusions

Overall our outcomes for a mixed ICU population with broad inclusion criteria for TH are comparable with those of published studies [2].

The use of intravascular cooling for TH was associated with minimal use of muscle relaxants allowing early neurological prognostication in our patient group.

However intravascular cooling to 32-34°C was associated with prolonged periods of rebound hyperthermia in a significant minority of patients (45.1%, mean time 8.6 hours).

We believe that TH to 32-34°C, using intravascular cooling, increases the risk of developing a rebound hyperthermia that could potentially exacerbate acquired neurological injury.

Our data supports the use of TTM to 36°C to mitigate any potential effect of rebound hyperthermia is this patient group.
